# Urinary markers of hydration during 3-day water restriction and graded rehydration

**DOI:** 10.1007/s00394-019-02065-7

**Published:** 2019-08-19

**Authors:** Evan C. Johnson, Ainsley E. Huffman, Hillary Yoder, Alberto Dolci, Erica T. Perrier, D. Enette Larson-Meyer, Lawrence E. Armstrong

**Affiliations:** 1 Human Integrated Physiology Laboratory, University of Wyoming, 1000 E. University Ave, Laramie, WY 82071 USA; 2 University of Utah School of Medicine, Salt Lake City, UT USA; 3 Department of Kinesiology, University of Alabama, Tuscaloosa, AL USA; 4 Danone Research, Palaiseau, France; 5 Department of Family and Consumer Sciences, University of Wyoming, Laramie, WY USA; 6 Hydration & Nutrition, LLC, Newport News, VA USA

**Keywords:** Hydration, Urine color, Urine osmolality, Hypohydration, Water recommendations

## Abstract

**Purpose:**

This investigation had three purposes: (a) to evaluate changes in hydration biomarkers in response to a graded rehydration intervention (GRHI) following 3 days of water restriction (WR), (b) assess within-day variation in urine concentrations, and (c) quantify the volume of fluid needed to return to euhydration as demonstrated by change in *U*_col_.

**Methods:**

115 adult males and females were observed during 1 week of habitual fluid intake, 3 days of fluid restriction (1000 mL day^−1^), and a fourth day in which the sample was randomized into five different GRHI groups: no additional water, CON; additional 500 mL, *G*_+0.50_; additional 1000 mL, *G*_+1.00_; additional 1500 mL, *G*_+1.50_; additional 2250 mL, *G*_+2.25_. All urine was collected on 1 day of the baseline week, during the final 2 days of the WR, and during the day of GRHI, and evaluated for urine osmolality, color, and specific gravity.

**Results:**

Following the GRHI, only *G*_+1.50_ and *G*_+2.25_ resulted in all urinary values being significantly different from CON. The mean volume of water increase was significantly greater for those whose *U*_col_ changed from > 4 to < 4 (+ 1435 ± 812 mL) than those whose *U*_col_ remained ≥ 4 (+ 667 ± 722 mL, *p* < 0.001).

**Conclusions:**

An additional 500 mL of water is not sufficient, while approximately 1500 mL of additional water (for a total intake between 2990 and 3515 mL day^−1^) is required to return to a urine color associated with adequate water intake, following 3 days of WR.

## Introduction

Adequate water intake is vital to health [1]. Most frequently dehydration (i.e., measured total body water loss) is associated with changes to exercise performance, thermoregulation, or even immune function [2, 3]. However, within the general population, the term “underhydration” has been defined as chronic low water intake accompanied by increased concentration of urine and fluid regulatory hormones, but not necessarily with a measurable decrement to total body water [4]. Defining underhydration is multidimensional, because the volume of water which is adequate for one individual can be substantially different from another due to extrinsic (e.g., environment) and intrinsic factors (e.g., physical activity, sodium, and caloric intake) [5]. Individualized water recommendations use urine concentration measurements [e.g., urine osmolality (*U*_osm_), urine-specific gravity (*U*_sg_), and urine color (*U*_col_)] as physiological criteria of water intake adequacy [6, 7]. The rationale for their use is that observation of concentrated urine is an indication of negative free water clearance which assists with maintenance of total body water. Within the focus of underhydration, urinary evaluation provides more value to researchers compared to markers of dehydration (i.e., plasma osmolality and body mass change). Plasma osmolality is limited when evaluating changes in 24-h water intake, because it is tightly regulated across a wide range of water intake volumes [8]. Body weight is less effective than urine evaluation, because it can fluctuate across days due to factors outside of water intake, such as caloric intake, particularly if measurements are greater than 1 day apart [9, 10]. Therefore, researchers have employed the *U*_osm_ threshold of 500 mOsm kg^−1^ within the general population as a reasonable target in the indication of adequate water intake, because *U*_osm_ accounts for factors such as body composition, dietary osmotic load, physical activity, and environment, and reflects the net result of urine concentrating and diluting mechanisms [11].

While *U*_osm_ and *U*_sg_ are capable of identifying different categories of water intake [12–14], the required technical skills and equipment are often confined to clinical or research settings. *U*_col_, on the other hand, is self-assessable [15] and has been correlated tightly with *U*_osm_ in previous studies [16, 17]. Furthermore, in adults and children, *U*_col_ alone offers the potential to identify underhydration [7, 15]. However, interpretation variation exists, because 24 h and single urine samples (i.e., spontaneous single samples) may be divergent [18]. Within single *U*_col_ sample analysis, interpretation could also be altered depending on the use of first morning [19] or early afternoon samples [20] as representation of adequate fluid intake. In addition, although the directionality of the *U*_col_ and water intake relationship is apparent; the full value of *U*_col_ has yet to be realized, because the volume of water that should be consumed to return to euhydration following a decrease in habitual water intake and the consequential underhydration or dehydration has not been defined.

Efforts have been made to quantify the volume of water necessary to change *U*_col_. A secondary, pooled analysis performed by Perrier et al. [21] combined studies in which daily total water intake (TWI) was manipulated, showing that an increase in TWI of 1110 mL day^−1^ [95% CI 914–1306] was required to lighten *U*_col_ by two units. Although useful, these results were limited, because (a) the volumes of TWI change that were pooled were generally large (i.e., > 1000 mL day^−1^), (b) the pooled sample populations were heterogeneous, and (c) the study only evaluated changes in *U*_col_ of two units which is not necessarily equivalent to an individual moving from above the *U*_col_ adequate water intake threshold of “4” on an eight-point scale [7], to below that threshold.

U_col_ thus appears to be particularly suited for applied self-evaluation outside of a research laboratory. However, inconsistencies in past investigations [19, 22, 23] as well as differentiation between dehydration and underhydration [4] point towards the need for additional and more specific research into the question of how to validly apply urinary markers to determine if, when, and how an individual has returned to adequate water intake following a period of insufficient intake. Therefore, the following investigation had three purposes: (a) to evaluate changes in hydration biomarkers in response to graded rehydration following 3 days of water restriction (WR), (b) assess within-day variation in urine concentration, and (c) quantify the volume of fluid needed to return to a urinary concentration associated with adequate water intake (i.e., a *U*_col_ of < 4 [7]) as demonstrated by change in *U*_col_. We hypothesized in reference to the above aims that (a) most participants would exhibit biomarkers indicative of underhydration following 3 days of WR and that there would be a progressive increase in the number of well hydrated individuals following the rehydration protocol, (b) urine concentration would be associated with the cumulative volume of water consumed over the progression of the day with the most concentrated occurring in the morning and least in the mid-day, and (c) at least 1000 mL of additional water would be needed to return and individual to *U*_col_ < 4.

## Subjects, materials, and methods

### Subjects

**Fig. 1:**
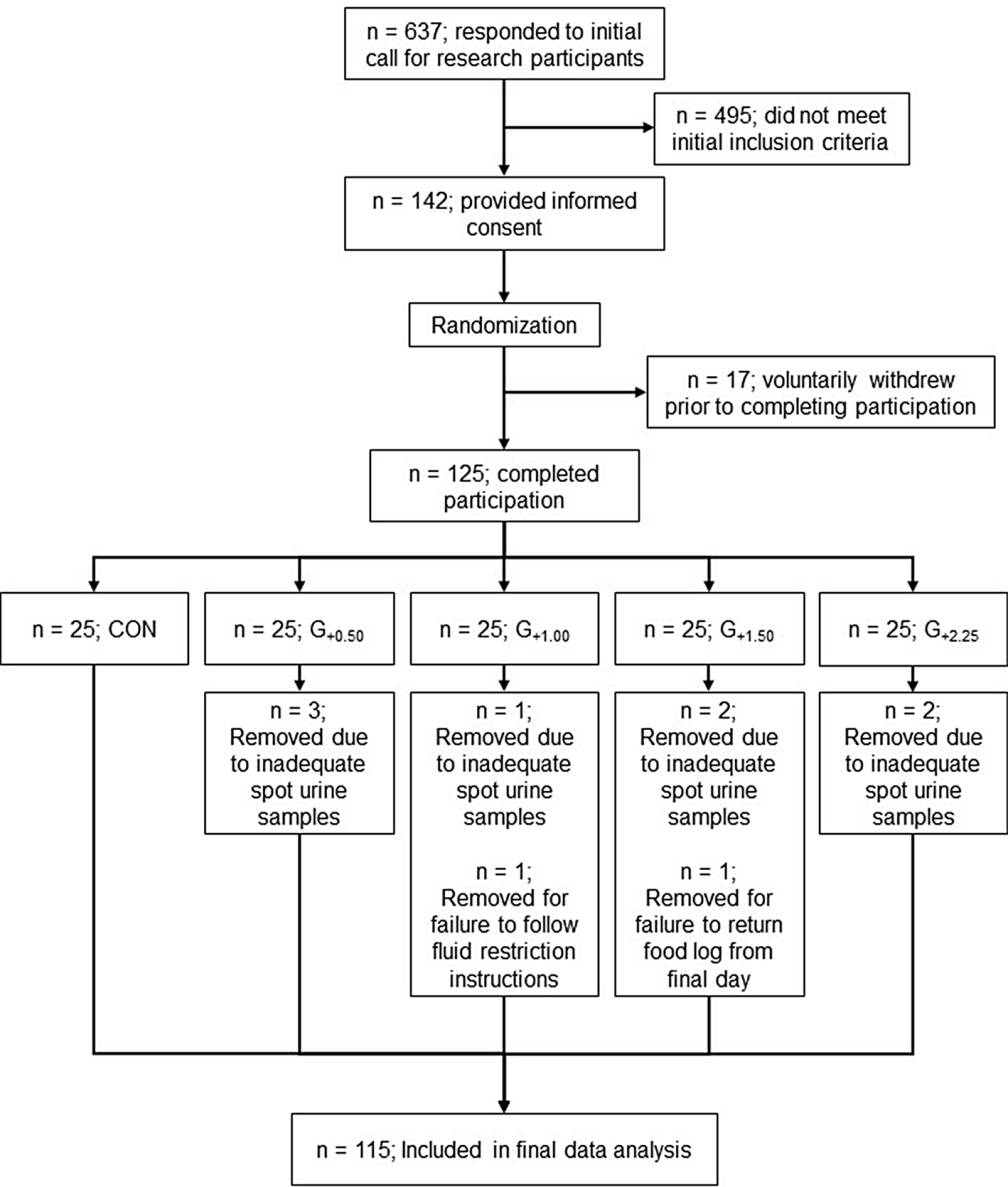
Flow diagram of subject recruitment

125 healthy men and women between the ages of 18 and 45 years participated in the current study. Of these, ten participants were excluded for the following reasons: failure to follow WR protocol (*n* = 1), failure to provide sufficient single urine samples (at least four of the six samples required by the protocol on all days; *n* = 8), and failure to return final dietary log (*n* = 1) (Fig. 1). Criteria for exclusion from this study included evidence of clinically relevant disease, regular prescription drug treatment that could disrupt fluid balance, body mass change (> 2.5 kg) or noteworthy dietary modifications in the past month, and exercising > 4 h per week. All of these factors were chosen, because they could potentially alter normal body water regulation. The University of Wyoming institutional review board approved this study protocol (protocol #20160524EJ01208), and all participants provided written informed consent in accordance with the Declaration of Helsinki. Each participant received financial compensation commensurate with the amount of time required for participation in the study.

### Study design

This study was an open-label, randomized-controlled trial, divided into two phases: “Baseline” which took place during week 1 where participants maintained normal eating and drinking habits, and “Experimental” which took place during week 2 where participants’ daily water intake was prescribed. During the experimental week, all participants were asked to refrain from traveling to a location that was more than 600 m different in elevation from Laramie, WY (elevation 2200 m), because abrupt changes in altitude may influence water balance [24]. Women’s participation in the study was not standardized for phase of menstrual cycle for two reasons. First, the purpose of this investigation was to provide ecologically valid recommendations for changes in water intake, which would not have been possible if all women participated during the identical phase of their menstrual cycle. Second, while the evidence of exogenous hormones influences on the set points for body fluid volume and tonicity, regulation is strong [25]. These changes do not appear to cause changes in total body water or renal free water clearance [26–28]. All participants completed five visits, two during Baseline and three during Experimental, to the Human Integrated Physiology Laboratory at the University of Wyoming. Outside of laboratory visits, participants recorded all food and fluid intake over the course of 12 consecutive days and collected 24-h urine samples on 4 days. The experimental conditions of the Baseline week were identical for all participants. The first 3 days of the Experimental week required all participants to limit their total fluid consumption to 1000 mL H_2_O day^−1^ (water restriction), with no other beverages, to elicit physiological water conservation consistent with elevated concentrations of the fluid conservation hormone vasopressin [29]. Participants received instructions as to what times during the day specific volumes of water were to be consumed. During 3 days of WR, the 1000 mL H_2_O day^−1^ was split into four equal 250 mL servings and consumed as follows: 250 mL with breakfast, 250 mL with lunch, 250 mL with dinner, and 250 mL before retiring to bed. On day 4 of Experimental week, participants were randomly assigned into one of the five groups of the graded rehydration intervention (GRHI): maintain 1000 mL H_2_O day^−1^ (CON group, 1000 mL H_2_O day^−1^ with no additional water) or increase their daily water intake corresponding to their randomly assigned group: additional 500 mL H_2_O day^−1^ (*G*_+0.50_), additional 1000 mL H_2_O day^−1^ (*G*_+1.00_), additional 1500 mL H_2_O day^−1^ (*G*_+1.50_), or additional 2250 mL H_2_O day^−1^ (*G*_+2.25_). Additional water was divided into three identical servings (e.g., *G*_+1.00_, 3 × 330 mL servings were given) and consumed at the following times, (1) additional morning water after the first single urine sample and at least 30 min before lunch, (2) additional afternoon water after the third single urine sample and at least 30 min before dinner, and (3) additional evening water after the fifth single urine sample and at least 30 min before retiring to bed. Single urine collection timing is described below.

### Experimental measurements

During the initial visit, participants completed an informed consent, medical history questionnaire, and had anthropometric measurements obtained, including body composition analysis with a DXA scanner (Lunar Prodigy; General Electric Healthcare). This initial visit marked the first day of food and fluid diary recording which continued throughout all 12 days of the study. On visits 2–5, participants returned the previous days’ 24-h urine sample to the laboratory for analysis. On visit three, four, and five, single samples were collected at six pre-determined times; (1) within 20 min after breakfast, (2) just before lunch and before 2nd 250 mL water, (3) within 20 min after lunch, (4) just before dinner and before third 250 mL water, (5) within 20 min after dinner, and (6) just before retiring to bed and before fourth 250 mL water. Each single sample was analyzed individually. Then, the specifically timed single samples were combined with voids that may have come between (which were collected in a single separate container), to reflect a complete 24 h collection. Any missing specimens (due to inability of participant to produce sample during WR) were indicated on the data sheet.

Each urine sample was analyzed for the following indices: *U*_osm_ using freezing point depression osmometry (Advanced Instruments Model 3250, Norwood, MA, USA), *U*_sg_ using manual refractometer (ATAGO T3-NE, Atago Corp., Japan), and *U*_col_ using the eight-point urine color scale (HydrationCheck.com) [30]. Urine specimens were excluded from urine color analysis if there was evidence of heavy menstrual bleeding (*n* = 1).

Participants recorded food intake using a 24-h diet diary on each day of the study, totaling 12 days. Fluid intake was separately recorded concurrently by participants using validated 24-h fluid diary [31]. Foods with high water content, such as soup, were not recorded in the fluid diary, but in the food diary. All information recorded in the fluid questionnaire and diet diaries were entered into the Nutritional Data System for Research software (Nutrition Coordinating Center, University of Minnesota, Minneapolis, MN, USA) to determine macronutrient, water, and sodium content. TWI was calculated by adding water from food to water intake from all beverages using data from both the diet and fluid diaries.

Due to the subjective nature of urine color assessment, all specimens had *U*_col_ assessed by a single researcher, under similar light conditions to avoid inter-rater differences in color ratings. In addition, an intra-rater reliability analysis was conducted to determine the precision of color ratings when confined to a single researcher. Thirty-one individual urine samples were presented to the *U*_col_ researcher in a random order and blinded by assigning letter and number combination codes. *U*_col_ of each of the 31 samples was assessed by the researcher on three separate occurrences 1 h apart, in between which the order of samples was re-randomized and assigned new identification codes.

### Statistical analyses

Sample size was calculated with the use of G*Power 3.0.10 (Franz Faul, Universitat Kiel, Germany) based on planned independent *t* tests between two means and the known volume change in water needed to lighten *U*_col_ by two units (power at 0.95, and *α* = 0.05). A sample size of 15 in each rehydration group was necessary to identify a meaningful difference of 1092 mL between groups, assuming an 877 mL standard deviation of the volume of water previously reported to lighten* U*_col_ by two units [21]. Based on previous studies and due to the time demands associated with data collection, we assumed that about 30% of the sample population either would choose to stop participation in the study, or would not adhere to all study requirements (e.g., incomplete dietary records). Therefore, we enrolled 25 participants in each rehydration group for a total of 125 study participants.

Data analysis was performed using, SPSS (IBM v24, Armonk, NY, USA). Intraclass correlation coefficient (ICC) analysis was used to determine intra-rater reliability for *U*_col_ assessment [32]. Cronbach’s *α* is representative of the lower bound internal consistency of a test or scale. ICC values less than 0.5 are indicative of poor reliability, values between 0.50 and 0.75 indicate moderate reliability, values between 0.75 and 0.90 indicate good reliability, and values greater than 0.90 indicate excellent reliability [33]. For purpose “A”, one-way ANOVAs were used to determine differences between demographic baseline variables for normally distributed variables, while Wilcoxon rank sum tests were used for non-normally distributed variables and Ucol because it is ordinal rather than continuous. Two-way repeated-measures ANOVAs [group (5) × time (3 or 2)] were used to establish main effects of time or group membership for all hydration-related variables. One test was performed for each variable between baseline and the end of the WR period. A second two-way ANOVA was performed between the end of WR and the day of the GRHI. Two ANOVAs were used for each variable, because the directionality of change would be expected to proceed in opposite directions between each of the above identified time courses. In the case of a statistically significant main effect of group, or interactions between group and time, post hoc independent samples *t* tests with Bonferroni corrections were used to define individual differences within *U*_osm_ and *U*_sg_, and post hoc Wilcoxon rank sum tests with correction for multiple comparisons were used for non-normally distributed variables and *U*_col_, between groups on visit 5 (i.e., the day of the GRHI). For purpose “B”, fluctuations in individual single urine osmolalities on the day of the GRHI were analyzed by two-way repeated-measures ANOVA [group (5) × time (6)]. Following significant interaction between group and time, post hoc independent samples *t* tests with Bonferroni corrections were used to define individual differences within *U*_osm_ at each time point. Finally, for purpose “C”, the return to adequate urine color was analyzed by separating out only those within the total sample whose 24 h *U*_col_ rose to > 4 (i.e., evidence of underhydration) on the third day of WR (*n* = 84), while those whose *U*_col_ remained ≤ 4 were eliminated from further analysis for purpose “C”. Next, during the GRHI, participants whose 24 h *U*_col_ dropped to < 4 were classified as adequately hydrated (*n* = 26), while those whose *U*_col_ remained ≥ 4 continued to be classified as underhydrated (*n* = 58). In an effort to perform the most conservative calculations, individuals with *U*_col_ = 4 were not classified as underhydrated following WR and were not classified as adequately hydrated following the GRHI, because *U*_col_ of 4 has previously been determined to be the criterion value between the classifications [7]. Change in TWI for all individuals was calculated by subtracting TWI on the final day of WR from TWI on the day of the GRHI. Change in TWI volume was compared between those who successfully moved from underhydrated to adequately hydrated due to the intervention versus those who remained underhydrated with an independent samples *t* test. Statistical significance was set a priori at *α* < 0.05, and all values are displayed as mean ± standard deviation unless otherwise noted.

## Results

### Baseline results

All participants’ baseline demographic and hydration information is shown in Table 1. No significant differences were observed between groups for any variables (all *p* > 0.093). The analysis of 31 individual urine specimens at three separate time points indicated high reliability of the *U*_col_ rater, Cronbach’s *α* = 0.993 (95% CI 0.987–0.996) *F*_[30,60]_ = 142.4, *p* < 0.001.

**Table 1:** Participant demographics

	CON	*G*_+0.50_	*G*_+1.00_	*G*_+1.50_	*G*_+2.25_	*p* value
*N* (%female)	25 (52%)	22 (50%)	23 (43%)	22 (55%)	23 (43%)	–
Age (years)	31 ± 9	31 ± 9	32 ± 8	31 ± 8	32 ± 9	0.997*
Height (cm)	174 ± 9	172 ± 9	177 ± 10	172 ± 11	171 ± 11	0.337
Weight (kg)	75.7 ± 17.9	73.8 ± 20.0	79.9 ± 14.8	70.8 ± 11.6	71.6 ± 22.4	0.152*
BMI (kg m^−2^)	25.1 ± 5.1	24.8 ± 5.1	25.6 ± 4.0	23.8 ± 2.7	24.1 ± 4.8	0.518*
Body fat (%)	28.5 ± 9.6	28.4 ± 7.8	29.9 ± 11.7	27.3 ± 8.4	25.4 ± 9.7	0.589
Urine osmolality (mOsm kg^−1^)	639 ± 299	555 ± 317	597 ± 271	566 ± 304	512 ± 353	0.617*
Urine color	4 ± 2	3 ± 2	5 ± 2	4 ± 2	4 ± 2	0.093*
Urine specific gravity	1.016 ± 0.008	1.014 ± 0.008	1.015 ± 0.007	1.015 ± 0.008	1.013 ± 0.010	0.652

*Wilcoxan rank sum test. All urine measurements taken from single urine samples provided during habitual dietary intake

### Water restriction

A significant main effect of time for TWI demonstrated that the WR protocol was successful in reducing all groups’ TWI from a grand mean of 3323 ± 1230 mL day^−1^ during the baseline observation period to 1730 ± 289 mL day^−1^ during the 3 days of WR (Table 2). Concomitantly, the grand mean of body mass significantly decreased (73.8 ± 16.3 to 73.4 ± 16.1 kg; *F*_[1,104]_ = 9.8, *p* < 0.001), while the grand means of all 24 h urinary markers of hydration increased; *U*_osm_ (465 ± 234 to 782 ± 213 mOsm kg^−1^; *F*_[2, 220]_ = 173.4, *p* < 0.001), *U*_col_ (3 ± 1 to 5 ± 1; *F*_[2, 216]_ = 127.5, *p* < 0.001), and *U*_sg_ (1.012 ± 0.006 to 1.021 ± 0.005; *F*_[2, 220]_ = 187.1, *p* < 0.001), as evidenced by significant main effects of time reported above for each of the variables. No significant main effects of group or interactions between group and time were observed. On the final day of WR, 84 of the 115 participants (73%) produced a 24 h urine sample with *U*_col_ > 4 (i.e., 5, 6, 7, or 8). This demonstrated that the WR protocol produced underhydration similarly in all five groups and that a large majority of participants were producing concentrated urine which was indicative of inadequate water intake.

**Table 2:** Water intake and corresponding markers of hydration

	Baseline	Water restriction Day 1	Water restriction Day 2	Water restriction Day 3		Post-graded rehydration intervention
Total water intake^a^ (mL day^−1^)						
CON	3382 ± 1286	1750 ± 277	1743 ± 260	1779 ± 335		1726 ± 297*
*G*_+0.50_	3248 ± 941	1700 ± 345	1681 ± 300	1820 ± 419		2205 ± 342*
*G*_+1.00_	3376 ± 1183	1721 ± 331	1784 ± 416	1758 ± 248		2676 ± 336*
*G*_+1.50_	3684 ± 1621	1734 ± 465	1661 ± 315	1794 ± 490		3118 ± 291*
*G*_+2.25_	2930 ± 989	1667 ± 250	1692 ± 260	1666 ± 188		3807 ± 184*
Main effect of group	0.426				< 0.001	
Main effect of time	< 0.001				< 0.001	
Interaction group × time	0.443				< 0.001	
Body mass^b^ (kg)						
CON	75.8 ± 18.1	–	–	75.8 ± 18.5		75.8 ± 18.2
*G*_+0.50_	71.7 ± 15.0	–	–	71.1 ± 14.5		71.3 ± 14.6
*G*_+1.00_	79.8 ± 14.6	–	–	79.4 ± 14.3		79.3 ± 14.6
*G*_+1.50_	70.8 ± 11.7	–	–	70.4 ± 11.7		70.4 ± 11.8
*G*_+2.25_	68.4 ± 14.3	–	–	67.8 ± 14.0		68.3 ± 14.3
Main effect of group	0.086				0.089	
Main effect of time	< 0.001				0.063	
Interaction group × time	0.584				0.232	
Urine osmolality (mOsm kg^−1^)						
CON	525 ± 291	–	810 ± 198	834 ± 223		873 ± 226^3,4,5^
*G*_+0.50_	474 ± 218	–	729 ± 247	839 ± 222		704 ± 254^4,5^
*G*_+1.00_	457 ± 205	–	700 ± 211	766 ± 255		613 ± 315^1^
*G*_+1.50_	416 ± 226	–	783 ± 207	793 ± 240		502 ± 250^1^
*G*_+2.25_	446 ± 219	–	769 ± 222	791 ± 268		452 ± 284^1,2^
Main effect of group	0.681				0.004	
Main effect of time	< 0.001				< 0.001	
Interaction group × time	0.881				< 0.001	
Urine color						
CON	4 ± 1	–	5 ± 1	5 ± 1		6 ± 1^3,4,5^
*G*_+0.50_	3 ± 1	–	5 ± 1	5 ± 1		5 ± 1^5^
*G*_+1.00_	3 ± 1	–	5 ± 1	5 ± 1		4 ± 2^1,5^
*G*_+1.50_	4 ± 1	–	5 ± 1	6 ± 1		4 ± 2^1^
*G*_+2.25_	3 ± 1	–	5 ± 1	5 ± 1		3 ± 2^1,2,3^
Main effect of group	0.495				0.001	
Main effect of time	< 0.001				< 0.001	
Interaction group × time	0.836				< 0.001	
Urine-specific gravity						
CON	1.014 ± 0.007	–	1.021 ± 0.005	1.022 ± 0.006		1.023 ± 0.006^4,5^
*G*_+0.50_	1.012 ± 0.005	–	1.019 ± 0.006	1.022 ± 0.006		1.019 ± 0.006^5^
*G*_+1.00_	1.012 ± 0.005	–	1.019 ± 0.005	1.020 ± 0.006		1.016 ± 0.007^1^
*G*_+1.50_	1.011 ± 0.006	–	1.021 ± 0.005	1.021 ± 0.006		1.014 ± 0.007^1^
*G*_+2.25_	1.012 ± 0.006	–	1.020 ± 0.006	1.021 ± 0.005		1.011 ± 0.008^1,2^
Main effect of group	0.648				0.003	
Main effect of time	< 0.001				< 0.001	
Interaction group × time	0.411				< 0.001	

All urinary measurements are from 24 h samples

^1,2,3,4,5^Wilcoxon rank sum post hoc test significantly different than CON, *G*_+0.50_, *G*_+1.00_, *G*_+1.50_, *G*_+2.25_, respectively

*Post hoc independent samples *t* test significantly different than all other groups at time point

^a^Baseline total water intake is average daily water intake over the 7 days of initial observation

^b^Body mass measurements occurred during the day. The body mass taken on the day of the graded rehydration intervention was only partially through the day; therefore, the body mass of the day after the graded rehydration intervention is reported here

### Increased water intake

Significant main effects of time, group, and a significant interaction between these factors demonstrated that the GRHI successfully changed each groups’ TWI. Post hoc *t* tests revealed that all groups’ TWI on the day of the GRHI were indeed significantly different from all other groups (all *p* < 0.001). Body mass did not significantly change in response to increased water intake. However, within urinary markers, main effects of time, group, and significant interactions between these factors demonstrated that makers related to water intake adequacy were changed by the water intervention (Table 2). Post hoc analyses revealed that typically groups whose rehydration intervention was only one intake volume step away (e.g., *G*_+0.50_ versus *G*_+1.00_) were not statistically different. At the end of the GRHI, 24 h urine samples differentiated both *G*_+1.50_ and *G*_+2.25_ from CON for all markers of hydration (Table 2).

**Fig. 2:**
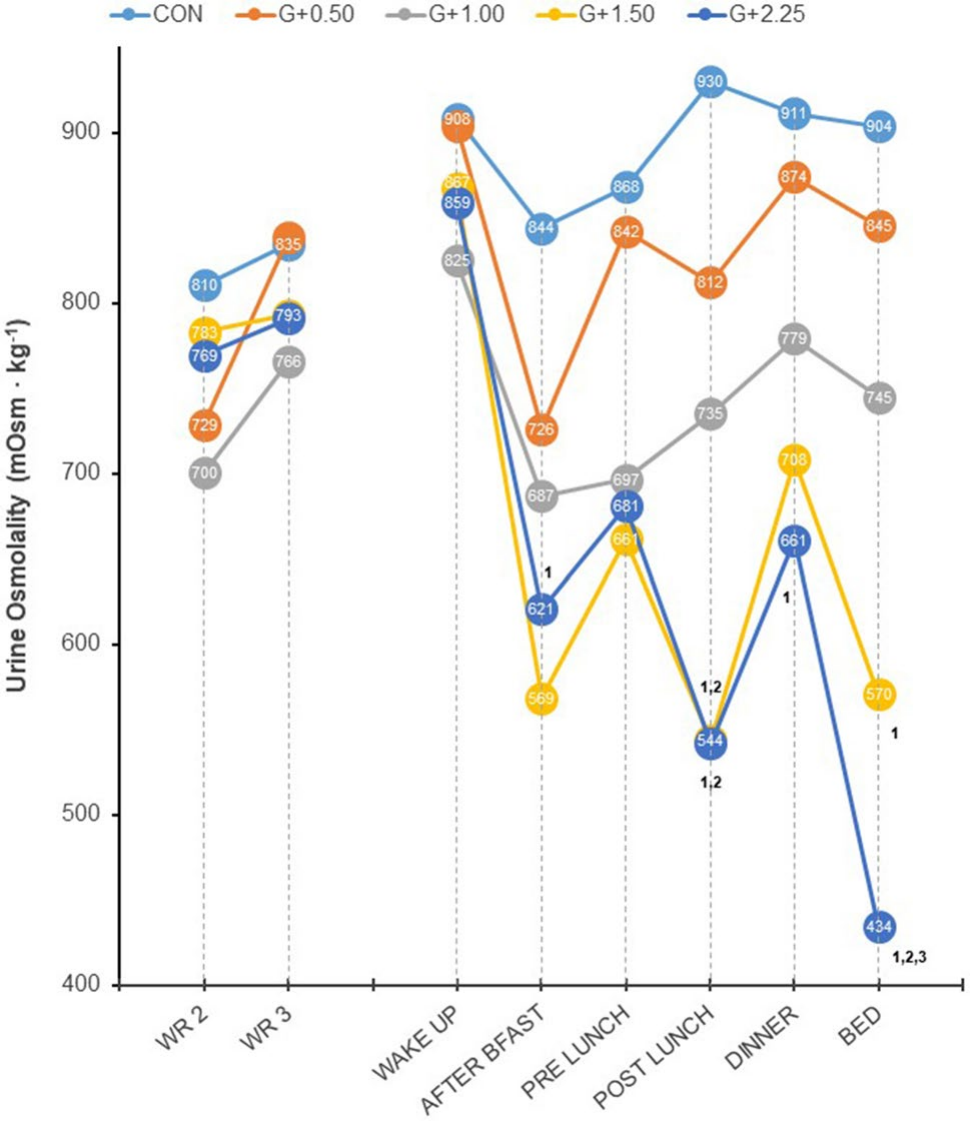
24-h urine osmolality for 2 final days of water restriction (WR) and single urine osmolality for six time points on day of graded rehydration intervention. ^1,2,3^Post hoc independent samples *t* test significantly different than CON, *G*_+0.50_, and *G*_+1.00_, respectively

Individual single urine samples collected on the day of the GRHI revealed that differences in 24 h *U*_osm_ were particularly attributable to individual time points within groups *G*_+1.50_ and *G*_+2.25_ (Fig. 2). A significant interaction (*F*_[4,108]_ = 2.767, *p* = 0.031) between group and time demonstrated that the GRHI changed *U*_osm_ differently between groups over the course of the day. Generally speaking, differences between groups tended to emerge later in the day. Specifically, post hoc tests showed that *G*_+1.50_*U*_osm_ was different from that of CON within single urine samples provided after breakfast, after lunch, and before bed, and different from *G*_+0.50_ after lunch only. Group *G*_+2.25_ was different from CON when samples were collected after lunch, after dinner, and before bed. In addition, *G*_+2.25_*U*_osm_ was significantly lower than *G*_+0.50_ after lunch and before bed, and lower than *G*_+1.00_ before bed (all *p* < 0.039, after corrections for multiple comparisons).

### Average amount of extra water needed to change UC from > 4 to < 4 in 24 h

**Fig. 3:**
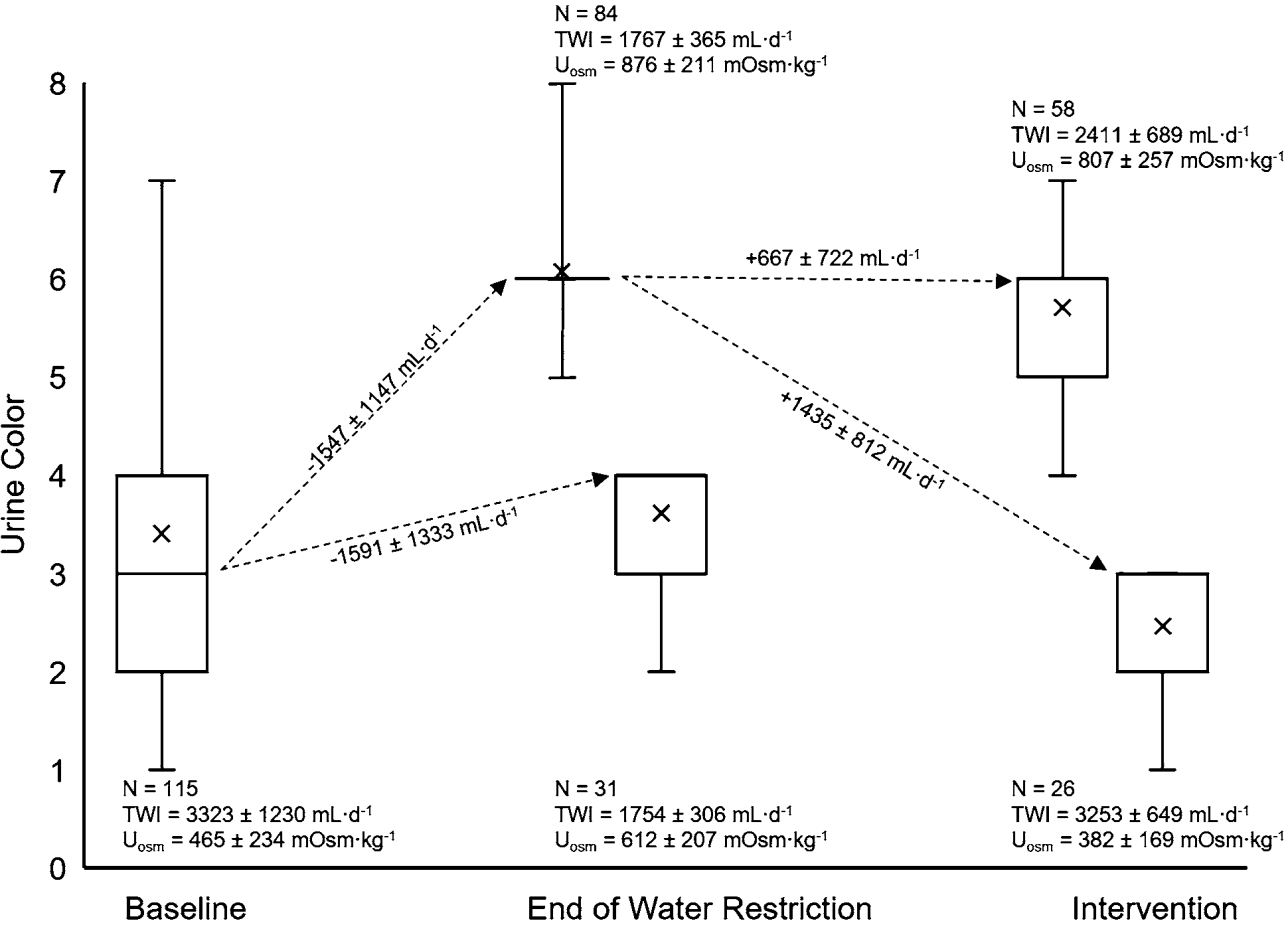
24-h urine color progression due to water restriction and graded rehydration intervention. The mean *U*_col_ for each grouping is represented by the “X” each box. The median is the middle line within each box. The 25th and 75th percentiles are represented by the top and bottom horizontal lines of each box. In some cases, the 25th percentile, median, and/or 75th percentile were equal due to the ordinal nature of *U*_col_. All dashed arrow lines are drawn to and from the median of each grouping. The range of each grouping is represented by the height of the error bars. Changes in TWI are indicated on each line between boxes with + and − signs indicating increased or decreased TWI, respectively

To assess the volume of water required to change from *U*_col_ > 4 to < 4, the effect of the GRHI was evaluated on those individuals whose *U*_col_ was > 4 after fluid restriction (Fig. 3). The individuals (*n* = 31) whose *U*_col_ did not exceed 4 during WR were eliminated from the following analyses. Of the 84 participants with *U*_col_ > 4 at the end of WR, only a minority (*n* = 26, or 30%) returned to *U*_col_ < 4 after the GRHI (Fig. 3). Those who returned to *U*_col_ < 4 more frequently came from the two subgroups with the highest water intake (19/26 from groups *G*_+2.25_ or *G*_+1.50_) than from the subgroups with lower water intake (7/26 from *G*_+1.00_ or *G*_+0.50_). On average, those who successfully returned to a urine concentration associated with adequate water intake (*U*_col_ < 4) consumed a TWI of 3253 ± 649 mL (TWI∆, 1435 ± 812 mL), while those who failed to return to adequate intake consumed significantly less (TWI, 2411 ± 689 mL; TWI∆ +667 ± 722 mL; both *p* < 0.001). Nineteen individuals in CON demonstrated *U*_col_ > 4 after 3 days of WR and none of these individuals reduced *U*_col_ < 4 on the GRHI day (for CON maintenance of 1000 mL H_2_O day^−1^ water intake). For each intervention group, 0%, 32%, 26%, 59%, and 65% for CON, *G*_+0.50_, *G*_+1.00_, *G*_+1.50_, and *G*_+2.25_, respectively, demonstrated *U*_col_ < 4 on the day of the GRHI.

## Discussion

The main findings of the current investigation were (a) 1000 mL day^−1^ of water administered over 3 days was sufficient to induce urinary markers consistent with inadequate fluid intake (i.e., elevated urine osmolality) in most, but not all participants and that approximately 1500 mL day^−1^ of additional water was required during the GRHI to reduce urine concentration below that of CON, (b) fluctuations in *U*_osm_ coincided with the timing and the compounded volume of additional water given on the day of the GRHI, and (c) an increase of water intake between 1107 and 1763 mL day^−1^, in addition to the baseline intake of 1000 mL day^−1^, is required to reduce an individual’s *U*_col_ from > 4 to < 4 within 24 h. These findings expand upon previous water intake investigations by allowing precise observation of the responses to graded rehydration and quantification of water volumes that are necessary to re-establish urinary concentration consistent with adequate water intake.

Following 3 days of WR, a majority of individuals displayed not only signs of inadequate water intake (i.e., elevated urine concentration), but those of dehydration (e.g., decrease of body mass). During the period of WR, individuals consumed only 1000 mL day^−1^ of plain water, and the remainder of their TWI (~ 700 mL day^−1^) came from food sources (e.g., fruit, soups, etc.). Our measurements did not include an estimation for metabolic water production. TWI was below both the U.S. National Academy of Medicine (3000–3700 mL day^−1^) [5] and the European Food Safety Authority (EFSA, 2000–2500 mL day^−1^) [34] adequate intake recommendations for females and males. The finding that most but not all individuals displayed signs of underhydration due to this WR provides two items of significance. First, this supports both above adequate intakes, because 1700 mL day^−1^ of TWI is not adequate among a selected sample of healthy individuals. However, it also demonstrates the variability in water needs across individuals. Even though the mean intake during baseline was nearly double the volume provided during WR, underhydration was not apparent in all individuals, which may be indicative of the extrinsic and intrinsic factors related to total water needs (e.g., environmental heat exposure or lean body mass, respectively).

The graded rehydration was important, because several past investigations specific to the relationship between water intake and hydration biomarkers have identified “high” and “low” drinkers, and then evaluated changes that occurred when TWI volumes are switched [12, 18, 35] (i.e., a change in TWI of > 1000 mL day^−1^). Although this approach is beneficial for evaluation of the largest and smallest TWI groups, its applicability is limited. It would be helpful to know if moderate increases in water intake could shift hydration biomarkers towards values associated with adequate water intake, because water interventions that impose large changes in TWI could be met with resistance by users. In this investigation, the group which received the smallest increase in water intake during the GRHI (*G*_+0.50_) realized no benefit due to the additional water, as evaluated by urinary biomarkers. This is similar to past research which found no differences in urinary or hematological biomarkers of hydration state after increasing chronic water intake over the course of 2 weeks from 35 to 40 mL kg^−1^ body mass (1685 ± 320 to 2054 ± 363 mL day^−1^) [36]. Although there may be physiological benefits of increasing water intake by volumes even as small as 100 mL, such as suppression of vasopressin secretion or exercise performance enhancement [37, 38], it is clear that urinary biomarkers of water intake cannot differentiate these small changes due to the relatively small samples sizes that were investigated.

In contrast to the lower volumes of GRHI that did not induce changes in urinary markers, the higher volumes resulted in significant changes. As mentioned above, approximately 1100 mL of additional water was previously recommended to decrease *U*_col_ by two units [21]. Our findings support these previous studies, because the low end of the 95% CI associated with moving from a *U*_col_ > 4 to < 4 was 1107 mL and the upper end of the 95% CI was 1763 mL. While this is a large range encompassing 600 mL (just over two 8 oz glasses), it reflects the range of *U*_col_ that was induced by the WR. In the previous study, the 1100 mL was associated only with a decrease of *U*_col_ by two units. This would only be applicable to the current investigation if that participant were moving from *U*_col_ = 5–3. The present findings also can be applied to an individual moving from *U*_col_ = 7 to *U*_col_ = 3. However, it is clear that even 2250 ml of additional water was not sufficient for some, as evidenced by the 35% of *G*_+2.25_ whose *U*_col_ remained ≥ 4 following GRHI. Because the GRHI took place over a single 24 h period, it is possible that a second day of increased water intake within many of the intervention groups may have allowed those participants to return to *U*_col_ < 4. Although from a different geographical population, the finding that free-living individuals with habitual TWI > 2500 mL day^−1^ were associated with optimal hydration [39] supports the theory that a longer duration of *G*_+1.50_ or *G*_+2.25_ GRHI would have been successful in returning a greater proportion to more desirable urine concentrations.

Factors such as (1) the relatively small gradations between the GRHI groups and (2) a sample population with a wide range of ages, body masses, and an equal distribution of both sexes make this investigation strong. However, there are also several limitations that are acknowledged. For example, the evidence of underhydration induced by WR over 3 days is a unique protocol. It is not clear if the urinary values associated with the WR or the GRHI would be similar if the alterations of fluid balance were induced over a shorter duration or as the result of chronic or habitual low intake. While the evidence of underhydration was strong, it was also assumed as evidenced by body mass measurements that dehydration was imparted by the WR protocol. However, the lack of increase in body mass following the GRHI, even in groups receiving higher volumes of water (e.g., *G*_+1.50_), makes it plausible that the − 0.4-kg change in body mass that was observed did not represent a loss of total body water and thus increased water intake would not influence body water. In this case, it is likely that the reduced urinary concentration markers were indicative of additional water excretion only and not changes in hydration status. This may be important, because both hydration status (i.e., total body water volume) and hydration process (i.e., daily water turnover) have been implicated in relationships with overall health [40]. It appears as if the present intervention is most relevant to the latter. A second limitation of this investigation is the self-report nature of the dietary logs. Finally, the limitations of self-report food logs are well documented, and thus, the reported values for macronutrient intake reported within this investigation should be viewed as an estimation.

The results of this investigation can be applied to a number of situations. For example, health care workers who demonstrate poor hydration practices [41] can be coached to evaluate their *U*_col_. In the case of noticing a darker *U*_col_ at the end of a work shift, the volume of additional water that should be consumed to achieve ideal urine concentration is now understood. Moreover, as increased water intake has been shown to significantly reduce the incidence of urinary tract infection (UTI) recurrence [42], these results may help young women who suffer from recurrent UTI to manage their fluid intake appropriately for secondary UTI prevention. Overall, the data of the present study provide a starting point regarding the optimization of water intake within populations known to exhibit increased urinary concentration, as well as within individuals seeking to improve their hydration status within a 24-h period.

## Abbreviations

CON: Control group, no additional water during rehydration phase
*G*_+0.50_: Additional 500 mL (0.50 L) water during rehydration phase
*G*_+1.00_: Additional 1000 mL (1.00 L) water during rehydration phase
*G*_+1.50_: Additional 1500 mL (1.50 L) water during rehydration phase
*G*_+2.25_: Additional 2250 mL (2.25 L) water during rehydration phase
GRHI: Graded rehydration intervention
TBW: Total body water
TWI: Total water intake, water from food, and beverages
TWI∆: Change in total water intake either between baseline and water restriction or between water restriction and the graded rehydration intervention
*U*_osm_: Urine osmolality (mOsmo kg^−1^)
*U*_vol_: Urine volume (mL)
*U*_sg_: Urine-specific gravity
*U*_col_: Urine color
WR: Water restriction

## References

[1] Popkin BM, D’Anci KE, Rosenberg IH (2010). Water, hydration, and health. Nutr Rev.

[2] Sawka MN, Montain SJ, Latzka WA (2001). Hydration effects on thermoregulation and performance in the heat. Comp Biochem Physiol A Mol Integr Physiol.

[3] Penkman MA, Field CJ, Sellar CM, Harber VJ, Bell GJ (2008). Effect of hydration status on high-intensity rowing performance and immune function. Int J Sports Physiol Perform.

[4] Kavouras SA (2019). Hydration, dehydration, underhydration, optimal hydration: are we barking up the wrong tree?. Eur J Nutr.

[5] Food and Nutrition Board, Institute of Medicine (2004). Dietary reference intakes for water, potassium, sodium, chloride, and sulfate.

[6] Manz F, Wentz A (2003). 24-h hydration status: parameters, epidemiology and recommendations. Eur J Clin Nutr.

[7] Perrier ET, Bottin JH, Vecchio M, Lemetais G (2017). Criterion values for urine-specific gravity and urine color representing adequate water intake in healthy adults. Eur J Clin Nutr.

[8] United States Department of Health and Human Services; Centers for Disease Control and Prevention; National Center for Health Statistics (1998) The Third National Health and Nutrition Examination Survey (NHANES III). Inter- University Consortium for Political and Social Research, Ann Arbor, MI, USA, 1998

[9] Cheuvront SN, Carter R, Montain SJ, Sawka MN (2004). Daily body mass variability and stability in active men undergoing exercise-heat stress. Int J Sport Nutr Exerc Metab.

[10] Chow CC, Hall KD (2008). The dynamics of human body weight change. PLoS Comput Biol.

[11] Perrier ET, Buendia-Jimenez I, Vecchio M, Armstrong LE, Tack I, Klein A (2015). Twenty-four-hour urine osmolality as a physiological index of adequate water intake. Dis Mark.

[12] Perrier E, Vergne S, Klein A, Poupin M, Rondeau P, Le Bellego L, Armstrong LE, Lang F, Stookey J, Tack I (2013). Hydration biomarkers in free-living adults with different levels of habitual fluid consumption. Br J Nutr.

[13] Armstrong LE, Pumerantz AC, Fiala KA, Roti MW, Kavouras SA, Casa DJ, Maresh CM (2010). Human hydration indices: acute and longitudinal reference values. Int J Sport Nutr Exerc Metab.

[14] Armstrong LE, Johnson EC, Munoz CX, Swokla B, Le Bellego L, Jimenez L, Casa DJ, Maresh CM (2012). Hydration biomarkers and dietary fluid consumption of women. J Acad Nutr Diet.

[15] Kavouras SA, Bougatsas D, Johnson EC, Arnaoutis G, Tsipouridi S, Panagiotakos DB (2017). Water intake and urinary hydration biomarkers in children. Eur J Clin Nutr.

[16] Armstrong LE, Maresh CM, Castellani JW, Bergeron MF, Kenefick RW, LaGasse KE, Riebe D (1994). Urinary indices of hydration status. Int J Sport Nutr.

[17] Armstrong LE, Soto JA, Hacker FT, Casa DJ, Kavouras SA, Maresh CM (1998). Urinary indices during dehydration, exercise, and rehydration. Int J Sport Nutr.

[18] Perrier E, Demazieres A, Girard N, Pross N, Osbild D, Metzger D, Guelinckx I, Klein A (2013). Circadian variation and responsiveness of hydration biomarkers to changes in daily water intake. Eur J Appl Physiol.

[19] Cheuvront SN, Munoz CX, Kenefick RW (2016). The void in using urine concentration to assess population fluid intake adequacy or hydration status. Am J Clin Nutr.

[20] Bottin JH, Lemetais G, Poupin M, Jimenez L, Perrier ET (2016). Equivalence of afternoon spot and 24-h urinary hydration biomarkers in free-living healthy adults. Eur J Clin Nutr.

[21] Perrier ET, Johnson EC, McKenzie AL, Ellis LA, Armstrong LE (2016). Urine colour change as an indicator of change in daily water intake: a quantitative analysis. Eur J Nutr.

[22] Hooper L, Bunn DK, Abdelhamid A, Gillings R, Jennings A, Maas K, Millar S, Twomlow E, Hunter PR, Shepstone L, Potter JF, Fairweather-Tait SJ (2016). Water-loss (intracellular) dehydration assessed using urinary tests: how well do they work? Diagnostic accuracy in older people. Am J Clin Nutr.

[23] Munoz CX, Johnson EC, Demartini JK, Huggins RA, McKenzie AL, Casa DJ, Maresh CM, Armstrong LE (2013). Assessment of hydration biomarkers including salivary osmolality during passive and active dehydration. Eur J Clin Nutr.

[24] Marriott BM, Carlson SJ, Institute of Medicine (US) Committee on Military Nutrition Research (1996). Nutritional needs in cold and in high-altitude environments: applications for military personnel in field operations.

[25] Stachenfeld NS (2008). Sex hormone effects on body fluid regulation. Exerc Sport Sci Rev.

[26] Cumberledge EA, Myers C, Venditti JJ, Dixon CB, Andreacci JL (2018). The effect of the menstrual cycle on body composition determined by contact-electrode bioelectrical impedance analyzers. Int J Exerc Sci.

[27] Hicks CS, McLester CN, Esmat TA, McLester JR (2017). A comparison of body composition across two phases of the menstrual cycle utilizing dual-energy X-ray absorptiometry, air displacement plethysmography, and bioelectrical impedance analysis. Int J Exerc Sci.

[28] Stachenfeld NS, Taylor HS, Leone CA, Keefe DL (2003). Oestrogen effects on urine concentrating response in young women. J Physiol.

[29] Armstrong LE, Johnson EC (2018). Water intake, water balance, and the elusive daily water requirement. Nutrients.

[30] Armstrong LE (2004) Hydration Check. Human hydration, LLC (Updated 27 July 2019). http://www.hydrationcheck.com/index.php. Accessed 10 Feb 2014

[31] Johnson EC, Peronnet F, Jansen LT, Capitan-Jimenez C, Adams JD, Guelinckx I, Jimenez L, Mauromoustakos A, Kavouras SA (2017). Validation testing demonstrates efficacy of a 7-day fluid record to estimate daily water intake in adult men and women when compared with total body water turnover measurement. J Nutr.

[32] Rankin G, Stokes M (1998). Reliability of assessment tools in rehabilitation: an illustration of appropriate statistical analyses. Clin Rehabil.

[33] Koo TK, Li MY (2016). A guideline of selecting and reporting intraclass correlation coefficients for reliability research. J Chiropr Med.

[34] EFSA Panel on Dietetic Products, Nutrition, and Allergies (2010). Scientific opinion on dietary reference values for water. EFSA J.

[35] Johnson EC, Armstrong LE (2013). Switching habitual small and large volume drinkers: outcomes and lessons learned. Nutr Today.

[36] Tucker MA, Adams JD, Brown LA, Ridings CB, Burchfield JM, Robinson FB, McDermott JL, Schreiber BA, Moyen NE, Washington TA, Bermudez AC, Bennett MP, Buyckx ME, Ganio MS (2016). No change in 24-hour hydration status following a moderate increase in fluid consumption. J Am Coll Nutr.

[37] Geelen G, Keil LC, Kravik SE, Wade CE, Thrasher TN, Barnes PR, Pyka G, Nesvig C, Greenleaf JE (1984). Inhibition of plasma vasopressin after drinking in dehydrated humans. Am J Physiol.

[38] Arnaoutis G, Kavouras SA, Christaki I, Sidossis LS (2012). Water ingestion improves performance compared with mouth rinse in dehydrated subjects. Med Sci Sports Exerc.

[39] Zhang N, Du S, Tang Z, Zheng M, Yan R, Zhu Y, Ma G (2017). Hydration, fluid intake, and related urine biomarkers among male college students in Cangzhou, China: a cross-sectional study-applications for assessing fluid intake and adequate water intake. Int J Environ Res Public Health.

[40] Perrier ET, Armstrong LE, Daudon M, Kavouras S, Lafontan M, Lang F, Peronnet F, Stookey JD, Tack I, Klein A (2014). From state to process: defining hydration. Obes Facts.

[41] El-Sharkawy AM, Bragg D, Watson P, Neal K, Sahota O, Maughan RJ, Lobo DN (2016). Hydration amongst nurses and doctors on-call (the HANDS on prospective cohort study). Clin Nutr.

[42] Hooton TM, Vecchio M, Iroz A, Tack I, Dornic Q, Seksek I, Lotan Y (2018). Effect of increased daily water intake in premenopausal women with recurrent urinary tract infections: a randomized clinical trial. JAMA Intern Med.

